# Effect of Dietary Modifications on Blood Pressure and Anthropometric and Biochemical Parameters in a Woman with Hypotension

**DOI:** 10.3390/jcm14134415

**Published:** 2025-06-20

**Authors:** Katarzyna Antosik, Damian Dyńka, Kinga Ziętara

**Affiliations:** Institute of Health Sciences, Faculty of Medical and Health Sciences, University of Siedlce, 08-110 Siedlce, Poland

**Keywords:** menu modification, diet, salt, potassium, coffee, water, hypotension, blood pressure, pulse

## Abstract

**Background**: Although abnormal blood pressure is predominantly associated with hypertension, the issue of hypotension remains insufficiently explored. Chronic asymptomatic hypotension, in particular, is rarely addressed in clinical nutrition research. This study aimed to evaluate the impact of targeted dietary modifications on blood pressure as well as selected biochemical, anthropometric, and lifestyle parameters in an individual with low baseline blood pressure. **Methods**: A single-subject observational study was conducted on a 23-year-old woman with chronic hypotension. Following a 4-week baseline period, a 4-week dietary intervention was implemented involving increased sodium and fluid intake, the introduction of coffee, and overall caloric enhancement combined with increased physical activity. Blood pressure was measured three times daily; anthropometric, biochemical, and lifestyle data were recorded weekly. **Results**: The intervention resulted in an increase in both systolic (from 93.76 to 101.21 mmHg) and diastolic (from 57.51 to 59.43 mmHg) blood pressure. The subject also reported improved energy levels, longer sleep duration, and reduced daytime fatigue. However, changes in anthropometric parameters were minimal and biochemical outcomes remained inconclusive. **Conclusions**: The findings suggest that dietary and lifestyle modifications may positively influence blood pressure and subjective wellbeing in individuals with hypotension. There is a clear need for further research focussing on the effects of dietary modifications on blood pressure parameters in individuals with hypotension.

## 1. Introduction

The influence of various dietary components on blood pressure has been extensively documented. Notable factors include sodium intake [1], dietary potassium [2], caffeine consumption through coffee [3], and the intake of ultra-processed foods rich in sugar, fat, and salt [4]. However, the majority of these studies focus on hypertension and its management. They commonly explore dietary interventions, such as DASH (Dietary Approaches to Stop Hypertension) and Mediterranean diets, that are associated with beneficial effects on blood pressure regulation [5,6,7]. At the same time, there are far fewer publications focussing on issues of the impact of dietary factors on people with hypotension.

Hypotension, or low blood pressure, may be defined as a decrease in arterial blood pressure below commonly accepted lower thresholds, generally ranging between 90/60 mmHg and 120/80 mmHg [8]. Although there is no universally accepted guideline for the classification of hypotension, these values are frequently cited in the literature. Specifically, hypotension is often described as a condition in which systolic blood pressure falls below 90 mmHg and/or diastolic pressure drops below 60 mmHg [9]. There are three main types of hypotension, i.e., chronic asymptomatic hypotension (observed in the woman in the present research) as well as orthostatic hypotension and hypotension of nervous origin [9]. Other types of hypotension include multiple system atrophy with orthostatic hypotension or postprandial hypotension [10]. Given the range of factors that influence hypotension and its different types and aetiologies, it is difficult to clearly define the epidemiology of hypotension [8].

The case of this woman seems particularly interesting for two main reasons. Firstly, the scientific literature on hypotension is much narrower compared with hypertension (which is mainly the focus of the recommendations) [11,12], therefore, exploration of this area is particularly valuable. There is a negligible amount of literature on the effect of diet and menu composition alone on changes in blood pressure parameters in people with hypotension (simultaneously covering changes in biochemical and anthropometric parameters). Secondly, the present study concerns a woman with so-called chronic asymptomatic hypotension, which in principle does not require treatment [13], although (as we found out in this study) even a simple dietary change can significantly affect blood pressure parameters, as described later in the article.

The aim of the study was to assess the effect of dietary modifications (mainly consisting of increased sodium intake with food, the introduction of coffee, and increased fluids and total energy) for 4 weeks (+4 weeks of observation and monitoring before the intervention) in correlation with increased physical activity on blood pressure parameters, selected biochemical and anthropometric parameters, and sleep duration in a 23-year-old woman with hypotension.

## 2. Materials and Methods

### 2.1. The Subject

A 23-year-old woman with hypotension who was underweight (BMI = 18 kg/m^2^, weight = 47.9 kg, and height of 163 cm) without any comorbidities and not taking medication was the subject of the study. The patient’s hypotension was observed at 17 years of age, but no pharmacological treatment was prescribed. The woman maintains a low level of physical activity (occasional walking and exercise), keeps her body weight relatively constant, and does not follow specific dietary patterns but tries to follow healthy eating rules. The woman does not drink coffee, does not smoke cigarettes, and consumes alcohol occasionally (once a month/two), regularly takes vitamin D (2000 IU) and zinc supplements, and sleeps five to six hours a day (with a bedtime between 23:00 and 24:00). As she reported, her wellbeing before taking part in the study varied (frequent headaches, weakness, and fatigue) and there were occasional sleep problems (difficulty falling asleep and waking up during the night).

The subject’s family history reveals a diverse blood pressure profile across both parental lines. Her mother has a history of hypertension, currently managed with pharmacological treatment. In contrast, her father consistently exhibits low blood pressure values, similar to the subject. On the paternal side, the grandmother is diagnosed with hypertension (treated), whereas the grandfather experiences chronic hypotension and takes medication to raise his blood pressure. On the maternal side, the grandmother had a history of treated hypertension and died due to a haemorrhagic stroke, while the maternal grandfather also suffers from hypertension, controlled with medication. The subject’s brother maintains normotensive values.

There were no symptoms indicative of autonomic nervous system dysfunction, such as dizziness, syncope, or postural hypotension. The subject’s blood pressure remained low across all time points without orthostatic variation and, therefore, orthostatic hypotension or other autonomic disorders were not clinically suspected. In terms of differential diagnosis, no features suggestive of inherited salt-wasting tubulopathies such as Gitelman or Bartter syndromes were observed. The subject’s biochemical profile at the baseline, combined with the absence of metabolic alkalosis or renal abnormalities, did not justify further genetic or hormonal testing. As such, no additional diagnostics in this area were performed within the scope of this case study.

### 2.2. The Diet Applied

For the first four weeks, the woman ate as usual and recorded her daily menu. In the subsequent 4 weeks, there was a modification of this menu, which was aimed at increasing sodium intake from food (from an average of 1601.09 ± 535.56 mg per day in stage I to 4400.02 ± 728.55 mg per day in stage II) and salt (from an average of 2.42 ± 1.16 g to 9.16 ± 1.83 g), the introduction of coffee (in the form of 1 cup (estimated at 102 mg caffeine)), and an increase in fluids (average from 2349.3 ± 226.65 mL per day in stage I to 3923.26 ± 508.02 mL per day in stage II) and energy (average from 1455.04 ±120.20 kcal per day in stage I to 1834.92 ± 98.62 kcal per day in stage II). In addition, the amounts of potassium (on average from 2936.06 ± 639.79 mg to 4358.58 ± 887.02 mg), magnesium (on average from 308.73 ± 81.84 mg to 488.73 ± 104.81), iron (on average from 10.73 ± 2.56 mg to 16.79 ± 3.55 mg), and other components were increased in stage II (relative to stage I), as shown in Table 1. During both stages of the diet, the woman consumed five meals a day (at 8:00, 11:30, 14:00, 17:30, and 20:30). The Kcalmar.pro programme was used to prepare the menu. Sample 3-day meal plans from stage I and stage II are presented in Table 2.

### 2.3. Physical Activity

The woman’s main physical activity both before and during the intervention was walking. During the first 4 weeks, the average daily number of steps was 4589.83 ± 852.38 (*p* = 0.01) and it increased to 8695.5 ± 1655.46 (*p* = 0.01) steps during the 4 weeks of intervention.

### 2.4. Blood Pressure Measurements

In both stage I and stage II of the study, blood pressure measurements were taken three times a day at around 07:30 (fasting, 30 min after waking up), around 15:30, and 22:30. Pulse was simultaneously measured with blood pressure (in parallel, at the same time and at the same frequency). Blood pressure and pulse were measured twice each time and the result was averaged. An ESPERANZA automatic upper-arm blood pressure monitor was used to measure blood pressure. Pulse measurements were taken using a Beurer Medical pulse oximeter (Beurer GmbH, Ulm, Germany).

### 2.5. Anthropometric Measurements

Once a week, body composition was analysed using the BIA (bioelectrical impedance) method, taking into account such parameters as body weight (in kg), height (in cm), body fat (in kg and %), muscle tissue (in kg and %), bone mass (in kg), total water content (in kg and %), lean mass (in kg), visceral fat (in pts), and BMI (kg/m^2^). Anthropometric measurements were taken using a TANITA DC-430 MA analyser (TANITA Corporation, Tokyo, Japan).

### 2.6. Biochemical Measurements

The following blood parameters were assessed: glucose, sodium, potassium, magnesium (stage II only), TSH (stage I only), and ferritin (stage II only). The tests were performed in the LuxMed Centre’s professional laboratory.

### 2.7. Statistical Elaboration of the Results

Tables and graphs were compiled in Microsoft Office 2016. STATISTICA 12 software was used to statistically process the results and the Student’s *t*-test for two independent samples and Pearson’s simple correlations were applied. A value of 0.05 was used as the significance level.

## 3. Results

### 3.1. Blood Pressure and Pulse (Before and During the Intervention)

**Figure 1:**
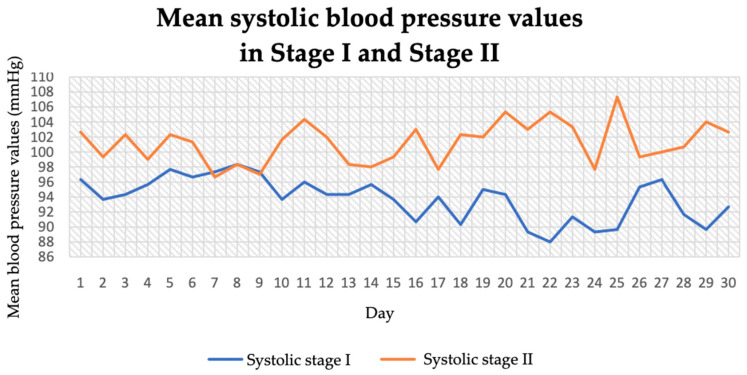
Mean systolic blood pressure values in stage I and stage II.

The dietary intervention applied improved systolic and diastolic blood pressure, regardless of the time of day the measurement was taken. When analysing systolic blood pressure values as an average measurement from the whole day, it was found that increasing sodium and salt in the diet and introducing more steps during the day increased this parameter by 7.45 mmHg units (Figure 1 and Table 3). In stage I, systolic blood pressure ranged between 88 and 97 mmHg. In contrast, in stage II of the study, it ranged between 97 and 107 mmHg. The average systolic pressure in stage I was 93.76 mmHg and in stage II it was 101.21 mmHg.

**Figure 2:**
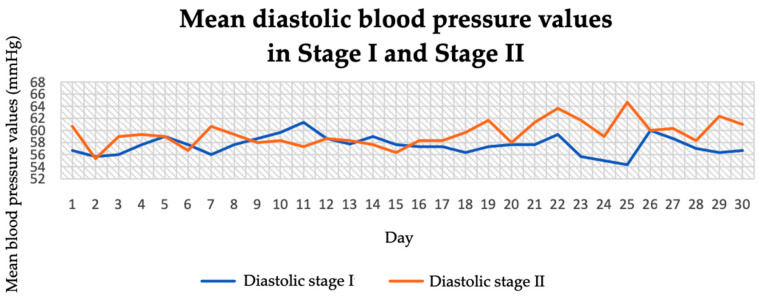
Mean diastolic blood pressure values in stage I and stage II.

In stage I of the study, diastolic blood pressure ranged between 54 and 61 mmHg (Figure 2). In stage II of the study, it ranged between 55 and 65 mmHg. The average diastolic pressure in stage I was 57.51 mmHg and in stage II it was 59.43 mmHg. The largest effect for systolic (increase of 9.5 mmHg units) and diastolic (increase of 2.5 mmHg units) measurements was observed for blood pressure measured at midday (104.07 ± 4.19/60.63 ± 3.14 mmHg in stage II of the study vs. 94.57 ± 3.72/58.13 ± 1.93 mmHg in stage I).

**Figure 3:**
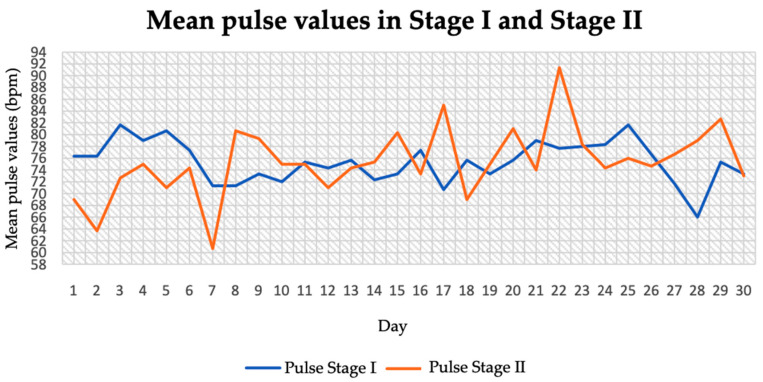
Mean pulse values in stage I and stage II.

The pulse values during examinations for both stages are shown in Figure 3. The patient generally recorded moderate, normal pulse values. In stage I, they ranged between 66 and 82 bpm (heartbeats/minute). In stage II of the study, the heart rate ranged between 60 and 92 bpm. The average pulse rate in stage I of the study was 75.36 bpm and in stage II it was 75.87 bpm. A slight increase in its value during the dietary intervention of 0.51 bpm units was shown.

A simple correlation analysis of the first stage of the study showed a significant association (at *p* ≤ 0.05) for systolic blood pressure with dietary protein (r = −0.40), total carbohydrates (r = 0.39), and folates (r= 0.38) (Table 4). When analysing the menus in stage I of the study, there was also a significant relationship between the pulse value and the fat (r = 0.50) and zinc (r = 0.39) content of the diet. In contrast, the dietary interventions applied in stage II of the study showed a significant relationship between pulse and dietary energy (r = 0.44), potassium (r= −0.49), calcium (r= −0.41), phosphorus (r= −0.54), iron (r= −0.38), and zinc content (r= −0.44). However, it should be emphasised that the obtained values of the correlation coefficients showed moderate correlations, which indicated that the demonstrated relationship between the parameters studied was not strong and requires further research on a larger population group.

### 3.2. Anthropometric Parameters

#### 3.2.1. Body Weight

**Figure 4:**
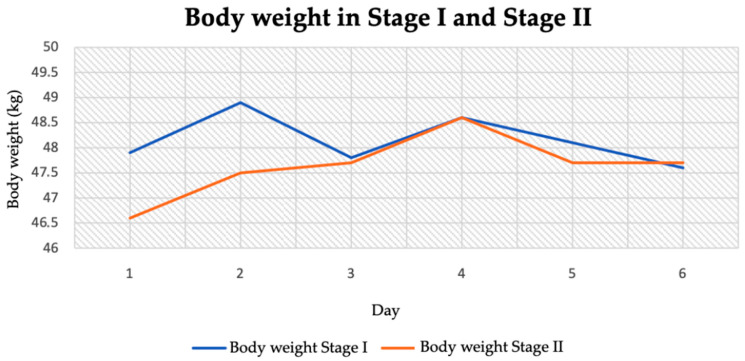
Body weight in stage I and stage II.

The patient’s body weight in stage I was not significantly different from that in stage II. The lowest body weight in stage I was 47.5 kg, while the highest was 49 kg. In contrast, in stage II of the study the lowest was 46.5 kg and the highest was 48.5 kg (Figure 4). The difference in mean body weight between the two stages was 0.52 kg (Table 4).

#### 3.2.2. Body Fat Mass

**Figure 5:**
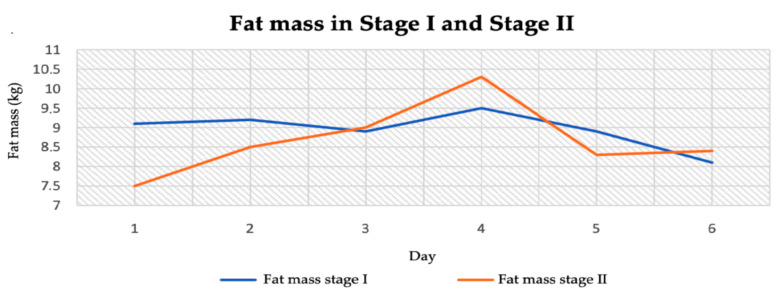
Fat mass content in stage I and stage II.

The patient’s adipose tissue was at a low level in both stage I and stage II. It averaged 8.95 kg in stage I and 8.67 kg in stage II (Table 4). The difference was 0.28 kg. The highest body fat content was recorded in stage II of the study (10.3 kg) (Figure 5).

#### 3.2.3. Muscle Tissue Mass

**Figure 6:**
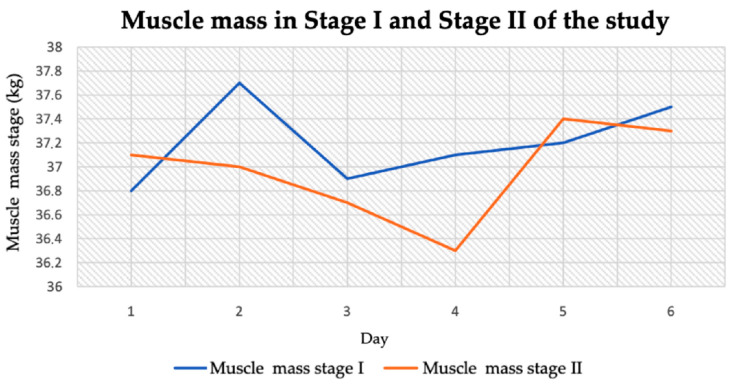
Muscle mass content in stage I and stage II.

The average muscle tissue content in stage I and stage II was similar. It was 37.2 ± 0.32 kg in stage I and 36.97 ± 0.37 kg in stage II (Table 4). The largest decrease in muscle mass was observed in one of the measurements in stage II, at which point it was 36.3 kg. The highest muscle mass content was recorded in stage I of the study and it was 37.7 kg (Figure 6).

#### 3.2.4. Lean Body Mass

**Figure 7:**
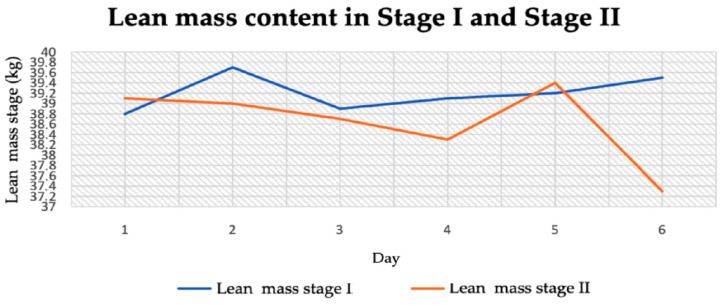
Lean mass content in stage I and stage II.

Lean body mass in stage I was between 38.8 and 39.8 kg (Figure 6). In stage II, on the other hand, it was 37.3–38.3 kg. The lowest lean body mass was recorded in stage II and it was 37.3 kg (Figure 7). The average lean body mass in stage I was 39.2 kg and in stage II it was 38.63 kg (Table 2). The difference between the stages was only 0.57 kg (Table 4).

#### 3.2.5. Body Water Content

**Figure 8:**
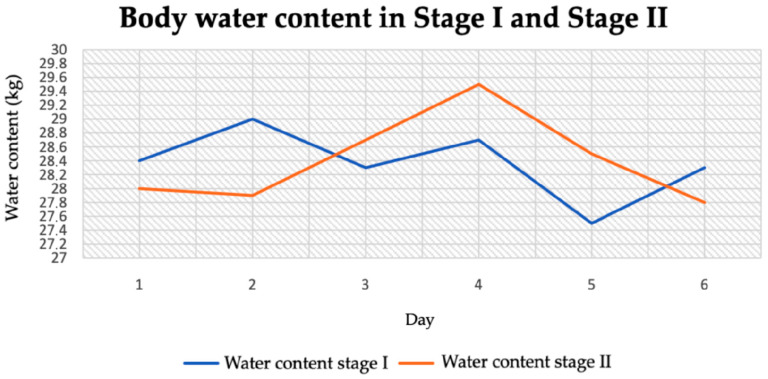
Body water content in stage I and stage II.

The average water content in stage I was 28.37 kg and in stage II it was 28.4 kg (Table 4). The difference between the stages was 0.3 kg. The lowest body water content was recorded in stage I (27.5 kg), while the highest was in stage II (29.5 kg) (Figure 8).

### 3.3. Biochemical Parameters

Venous blood samples were collected by qualified medical personnel in the morning, following an overnight fast. The analyses were performed at the LuxMed Centre laboratory, a certified clinical facility, using standard automated methods for each parameter in accordance with the manufacturer’s protocols and laboratory quality-control procedures. The determinations performed in both stages included glucose, potassium, and sodium. Blood sodium levels, despite the increased salt in the diet, did not dramatically change. For stage I, the value was 140 mmol/L, while for stage II it was 138 mmol/L (a difference of 0.2 mmol). Similarly, the potassium level before the start of stage I was 4.7 mmol/L and at the end of stage II it was 4.1 mmol/L (so there was a decrease). Fasting glucose levels decreased from 94 mg/dl before stage I to 87 mg/dl after stage II.

### 3.4. Length of Sleep

**Figure 9:**
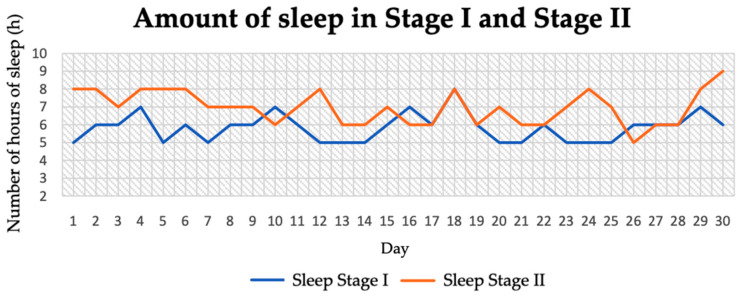
Amount of sleep in stage I and stage II.

The average amount of sleep in stage I was 5.83 ± 0.78 h, while in stage II it was 6.97 ± 0.95 h. In stage II of the study, the amount of sleep increased by 1.14 h (Table 3). Figure 9 shows the length of sleep duration each day in both stages of the diet.

### 3.5. Quality of Life

Following the introduction of the dietary intervention and lifestyle changes, the patient observed an improvement in her wellbeing. Observations reported by the woman included reduced daytime sleepiness, more energy during the day, reduced dark circles under the eyes, and a reduced perception of cold.

## 4. Discussion

The increase in systolic blood pressure (from an average of 93.76 mmHg to 101.21 mmHg) and diastolic blood pressure (from an average of 57.51 mmHg to 59.43 mmHg) for a patient with hypotonia can be considered to be favourable. Changes in these parameters correlated with the intervention in the form of an increase in the intake of water, coffee, and sodium-containing products, which automatically also increased the intake of potassium, magnesium, iron, and total dietary calories (from both protein, fat, and carbohydrates), among others. In the context of an increase in sodium intake, the observed correlation with an increase in blood pressure parameters is reflected in the available studies. An increase in blood pressure is considered to be the primary effect of a sodium-rich diet, which is considered unfavourable in most cases as the problem of hypertension is incomparably greater than that of hypotension. Therefore, in common recommendations and current findings, the recommended maximum amount of sodium (e.g., by the World Health Organization) is <2000 mg per day, corresponding with <5 g salt per day [14]. Available meta-analyses confirm the almost-linear correlation of increased sodium intake with the risk of hypertension in cohort studies, confirming sodium restriction in both hypertensive and normal blood pressure subjects [15]. A meta-analysis in 2024 showed that for every 100 mmol reduction in 24 h urinary sodium excretion, systolic blood pressure decreased by an average of 6.81 mmHg and diastolic blood pressure by an average of 3.85 mmHg. The authors observed a dose–response relationship between sodium reduction and blood pressure in treated patients with hypertension [16]. This is also confirmed by randomised controlled trials (RCTs); for example, in one, the authors showed significantly higher systolic blood pressure in a group consuming 12 g of sodium chloride compared with 6 g (121 ± 4 mmHg vs. 115 ± 4 mmHg) [17]. Another RCT showed significantly higher average arterial pressure under conditions of increased salt intake of up to 15 g per day [18]. In a randomised controlled trial of 2024, the authors showed that lowering sodium intake in just 24 h led to a reduction in blood pressure by an average of 9/5 mmHg compared with the control group [19]. It is worth noting, however, that not all authors agree on the cause-and-effect relationship between sodium intake and increased blood pressure, and that an increase in blood pressure may somehow be a result of an increased intake of sugars and fats (of which salty products have a higher content), rather than being due to sodium intake per se [20]. What is noteworthy, however, is that in our study, the woman, despite a significant increase in the intake of sodium-containing products (from an average of 2.42 ± 1.16 g per day in stage I to 9.16 ± 1.83 g per day in stage II), did not achieve an increase in blood sodium values and, in fact, there was a reduction (from 140 mmol/L in stage I to 138 mmol/L in stage II). The same was true for potassium, where the woman’s intake increased on average from 2936.06 ± 639.79 mg in stage I to 4358.58 ± 887.02 mg in stage 2, yet blood potassium values decreased from an average of 4.7 mmol/L to 4.1 mmol/L. It is possible that the increase in the woman’s blood pressure values was influenced by a reduction in potassium concentration as it is well known that a reduction in blood potassium concentration is associated with an increase in blood pressure. One study found significant negative correlations for blood potassium levels with blood pressure values [21]. It is, therefore, not surprising that hypertension is more common in patients with hypokalaemia [22]. It is worth noting, however, that increases in sodium and potassium intake were not the only changes and other factors may have influenced the concentration of these electrolytes as well as blood pressure per se, such as an increase in fluid intake, coffee, or the introduction of physical activity.

The increase in coffee consumption in the woman also probably significantly contributed to the blood pressure values. The effect of coffee on raising blood pressure is a widely researched phenomenon, although the research is inconclusive. Coffee is known to be a rich source of caffeine, which is an antagonist of A1 and A21 adenosine receptors, which have a transient effect on raising blood pressure. In a publication by Mesas et al., the authors note that the administration of 200–300 mg of caffeine resulted in a mean increase in systolic blood pressure of 8.1 mmHg and diastolic blood pressure of 5.7 mmHg [23,24]. Importantly, however, it appears that regular coffee consumption does not increase blood pressure, as shown in a 10-year prospective study of 2024. The authors concluded that habitual coffee consumption is neutral to blood pressure values measured at home, in the office, and in outpatient settings [25]. It was even noted in a systematic review with a meta-analysis in 2023 that there is an inverse relationship between coffee consumption and risk of hypertension [26]. It is worth noting that, in our study, the woman did not drink coffee until the intervention was introduced, so blood pressure in a person unaccustomed to consuming caffeine in this form may indeed rise periodically (as confirmed by the above publications, among others). At the same time, it is likely that the continued regular consumption of coffee by the woman would not cause an increase in her blood pressure. It is worth noting at this point that the introduction of coffee consumption may have exacerbated diuresis (along with the increased fluid intake), which translated into decreased serum potassium concentrations despite the increased consumption. The literature examines this phenomenon in detail, and the reduction in potassium concentration as a result of coffee consumption is widely reported in publications. For example, there was a case of a woman who even developed coffee-induced hypokalaemia [27] as well as one of a 29-year-old man who was admitted to hospital as a result of high coffee intake due to symptoms of hypokalaemia [28]. The findings are not surprising, given that there is a negative correlation between blood potassium and caffeine concentrations [29]. Therefore, the increase in blood pressure in the woman in our study may have been due to a reduction in blood potassium concentration, which was due, among other things, to the effect of the coffee introduced into the diet.

An increase in the woman’s fluid intake from an average of 2349.3 ± 226.65 to 3923.26 ± 508.02 mL per day may also have had an impact on blood pressure values. This is because it is known that an increase in fluid intake will increase blood pressure, as shown, for example, in the study by Buchineni et al. The authors demonstrated that in healthy adults, there was a significant increase in systolic blood pressure (from 113.6 ± 6.2 mmHg at 0 h to 127.7 ± 9.6 mmHg at 15 min and 120.7 ± 5.5 mmHg at 75 min) and diastolic blood pressure (from 73.6 ± 6.2 mmHg to 85.9 ± 7.4 mmHg at 30 min) after consuming 2000 mL [30]. The authors of another randomised controlled trial showed that two litres of additional water consumed for 2 weeks significantly increased daytime blood pressure and reduced feelings of dizziness in healthy individuals [31], which perfectly corresponds with our observations. The woman in our study increased her daily water intake by an average of 1575 mL (which is similar to the 2 litres from the Jormeus et al. study) for 4 weeks (compared with 2 weeks) and also increased her blood pressure values while also improving her quality of life in terms of less fatigue and sleepiness and more energy throughout the day, compared with the results of Jormeus et al. in which there was an improvement in quality of life in terms of, among other things, reduced dizziness. At the same time, an increase in fluid intake must result in an increase in urinary fluid excretion, and, knowing of the loss of potassium in urine [32], it can be assumed that this could have been one of the mechanisms for the decrease in serum potassium concentrations and, therefore, also one of the potential mechanisms for the increase in blood pressure in the woman.

The lack of significant differences in anthropometric parameters reflects the short intervention period (4 weeks) and the relatively small increase in physical activity as this was mainly the introduction of more daily steps (from 4589.83 ± 852.38 to 8695.5 ± 1655.46 on average) and, importantly, baseline body composition (the woman was already low in body fat at the baseline). Also, the literature shows no significant difference in anthropometric parameters according to the number of steps, as demonstrated in the randomised study by Bailey et al. The authors concluded that there was no significant difference between female students walking 10,000 steps, 12,500 steps, and 15,000 steps in terms of weight gain or body fat [33]. Some studies show some correlation, e.g., Thomson et al. observed that an increase in the daily step count by 1000 resulted in a 0.1 per cent decrease in body fat [34], although one should be extremely cautious in this context as body composition is significantly influenced by factors other than simply introducing a step count without monitoring diet or other physical activity.

Improvements in quality of life can be attributed to a number of factors that changed in stage II. Firstly, an increased step count can significantly affect quality of life and health status (which is also associated with quality of life), as confirmed by several studies [35,36,37]. The same applies to sleep quality, as the step count has been shown to be positively correlated with sleep efficiency and inversely correlated with wakefulness time after sleep [38]. Another study found that increasing the number of daily steps reduces daytime stress and sleepiness and increases sleep efficiency, but does not increase sleep duration [39]. Simply nourishing the body with more protein, fats, carbohydrates, vitamins, and micronutrients could also have an impact on quality of life and length of sleep.

In summary, the dietary modifications introduced in this case—most notably, increased sodium and fluid intake, the inclusion of coffee, and higher total energy consumption—were associated with an increase in both systolic and diastolic blood pressure, improved subjective energy levels, and longer sleep duration. These findings support the hypothesis that even simple, non-pharmacological interventions can positively influence physiological outcomes in individuals with chronic hypotension. Given the non-invasive nature of the intervention and the absence of adverse outcomes, the potential benefits, such as improved blood pressure regulation and enhanced wellbeing, were considered to outweigh any minimal risks involved in this case.

Although this study was based on a single patient, the findings indicate that simple, non-pharmacological strategies, such as an increased intake of sodium and fluids, moderate coffee consumption, and higher caloric intake, may contribute to improved blood pressure regulation in individuals with chronic asymptomatic hypotension. Such measures might be particularly useful in patients experiencing decreased vitality, excessive tiredness, or reduced daily functioning. Provided that these interventions are monitored by healthcare professionals, they could serve as a practical and low-risk complement—or, in some cases, an alternative—to pharmacological therapy. The outcomes observed in this case underscore the potential value of further investigations into lifestyle and dietary approaches in the clinical management of hypotension.

## 5. Limitations

There are several limitations that should be taken into account when interpreting the findings of this study. As the data were collected from a single individual, the results cannot be readily generalised to the broader population of individuals with hypotension, particularly those with different clinical backgrounds or comorbidities. The participant demonstrated a high level of health awareness and closely adhered to the dietary and lifestyle recommendations, which likely contributed to the reliability of the collected data. However, this level of engagement may not be typical for most patients and may introduce adherence-related bias, potentially amplifying the observed effects. Although some information on the family history of blood pressure variations was available, no detailed genetic assessment was performed, limiting insights into hereditary influences. Additionally, as with any observational design, the presence of unmeasured confounding factors could not be entirely excluded.

## 6. Conclusions

The results provide important insights into the impact of dietary intervention in a woman with hypotension. The extremely small number of studies among the population of people with hypotension makes the present study somewhat unique.

Most of the results obtained demonstrated a positive effect of the dietary modifications on the 23-year-old woman, which manifested, among other things, as an increase in blood pressure (which was beneficial in this case), an increase in sleep duration, a reduction in daytime sleepiness, more energy during the day, reduced dark circles under the eyes, and a reduction in perceived cold. The intervention did not significantly affect the values of the anthropometric parameters.

Until data from a larger cohort of patients is collected, drawing definitive conclusions from the present study remains limited. There is a clear need for randomised controlled trials that can provide more robust and reliable evidence than a single observational case study. Nevertheless, the findings obtained represent a meaningful step toward further exploration of this topic and may serve as a valuable starting point for future, more comprehensive investigations. The aim of this is to develop the most effective dietary strategies and lifestyle modifications that may contribute to improving the health and quality of life of individuals struggling with hypotension.

## Figures and Tables

**Table 1:** Nutritional value and content of selected dietary components in the first and second stage of the study.

	Menu, Stage I	Menu, Stage II	Student’s *t*-Test	
	Average and Standard Deviation	Average and Standard Deviation	*t*	*p*
Energy (kcal)	1455.04 ± 120.20	1834.92 ± 98.62	−13.16	0.01
Protein (g)	75.61 ± 11.84	97.04 ± 13.18	−6.51	0.01
Fat (g)	47.7 ± 6.51	63.74 ± 7.82	−8.49	0.01
Carbohydrates (g)	170.5 ± 21.2	204.13 ± 26.87	−5.29	0.01
Sodium (mg)	1601.09 ± 535.56	4400.02 ± 728.55	−16.67	0.01
Potassium (mg)	2936.06 ± 639.79	4358.58 ± 887.02	−7.00	0.01
Calcium (mg)	676.84 ± 296.07	879.52 ± 277.11	−2.69	0.01
Phosphorus (mg)	1516.25 ± 2211.3	1538.85 ± 205.25	−0.05	0.96
Magnesium (mg)	308.73 ± 81.84	488.73 ± 104.81	−7.29	0.01
Iron (mg)	10.73 ± 2.56	16.79 ± 3.55	−7.46	0.01
Zinc (mg)	7.45 ± 1.99	10.97 ± 2.52	−5.90	0.01
Folate (µg)	348.86 ± 107.1	533.54 ± 139.62	−5.65	0.01
Vitamin B12 (µg)	2.47 ± 1.1	4.21 ± 2.5	−3.42	0.01
Vitamin C (mg)	175.74 ± 84.06	263.81 ± 137.38	−2.94	0.01
Vitamin D (µg)	2.44 ± 2.74	5.18 ± 4.74	−2.69	0.01
Saturated fatty acids (g)	11.79 ± 3.7	14.83 ± 3.55	3.20	0.01
Monounsaturated fatty acids (g)	18.59 ± 4.37	26.91 ± 5.3	−6.52	0.01
Polyunsaturated fatty acids (g)	9.66 ± 3.1	12.82 ± 3.70	−3.52	0.01
Cholesterol (mg)	226.05 ± 155.81	345.48 ± 134.05	−3.13	0.01
Water (g)	2349.3 ± 226.65	3923.26 ± 508.02	−15.24	0.01
Salt (g)	2.42 ± 1.16	9.16 ± 1.83	−16.74	0.01
Glucose (g)	10.43 ± 5.45	13.79 ± 6.11	−2.21	0.04
Fibre (g)	24.15 ± 6.61	32.18 ± 5.50	−5.03	0.01

*t*: Student’s *t*-test value; *p*: significance level, significant at *p* ≤ 0.05.

**Table 2:** Sample 3-day meal plans from stage I and stage II.

	Menu, Stage I			Menu, Stage II		
	Day I	Day II	Day III	Day I	Day II	Day III
Breakfast	Whole eggs, 120 g Rapeseed oil, 5 g Tomato, 100 g Spelt bread, 50 g Ketchup, 5 g	Graham roll, 53 g Plant-based butter, 2 g Turkey ham, 22 g Semi-fat salami cheese, 5 g Cherry tomatoes, 20 g Green tea, 250 mL	Green tea, 500 mL Eggs, 56 g WPC protein powder, 15 g Wheat flour, 45 g Banana, 120 g Peanut butter, 10 g	Graham bread, 60 g Plant-based butter, 5 g Edam cheese, 15 g Tomato, 50 g Salt, 2 g Green tea, 900 mL Black coffee, no sugar, 250 mL	Green tea, 250 mL Black coffee, no sugar, 250 mL Spelt bread, 60 g Peanut butter, 15 g Banana, 120 g Salt, 2 g	Oatmeal flakes, 50 g Banana, 120 g Chicken eggs, 56 g Natural bee honey, 12 g Dark chocolate, 12 g Baking powder, 3 g Salt, 2 g Green tea, 250 mL Black coffee, no sugar, 250 mL
Brunch	Banana, 120 g Vanilla Skyr yogurt, 150 g Water, 500 mL	Wholegrain roll, 65 g Plant-based butter, 3 g Turkey ham, 22 g Semi-fat salami cheese, 5 g Tomato, 22 g Red bell pepper, 6 g Apple, 200 g	Millet flakes, 50 g Eggs, 112 g Banana, 75 g Water, 1000 mL	Mineral water, 2000 mL Banana, 120 g Cocoa, 10 g Milk 2%, 250 mL	Mineral water, 2250 mL Oats, 30 g Oat bran, 14 g Cottage cheese, 50 g Milk 2%, 80 mL Eggs, 112 g Raspberries, 50 g Banana, 50 g	Rice waffles with muesli, 30 g Raspberries, 60 g Blueberries (American), 50 g Milk 1.5% fat, 125 mL Whey protein concentrate (WPC), 30 g
Lunch	Chicken breast cutlet, 133 g Boiled potatoes, 120 g Pickled cucumbers, 80 g Grated carrot salad, 102 g	Boiled potatoes, 120 g Turkey breast cutlet, 133 g Warm beets, 213 g Water, 500 mL	Chicken breast fillet, 100 g Potatoes, 200 g Cucumber, 150 g Sour cream 10%, 70 g	Wheat flour, 55 g Eggs, 56 g Baking powder, 3 g Milk 2%, 50 mL Rapeseed oil, 5 g 100% jam, 30 g Peanut butter, 15 g Salt, 2 g	Chicken breast, 150 g White rice, 60 g Stir-fry vegetables, 200 g Salt, 3 g	Barley groats (pearl barley), 60 g Fresh salmon, 100 g Pickled cucumber, 60 g Tabasco sauce, 10 g Salt, 3 g
Afternoon Tea	Apple, 200 g Unsalted cashew nuts, 15 g Apple juice, 375 mL	Light cottage cheese, 150 g Graham roll, 65 g Blueberries, 50 g Cinnamon, 3 g	Pineapple, 150 g	Natural yogurt, 250 g Kiwi, 150 g Walnuts, 15 g Corn flakes, 40 g	Raisins, 30 g Dark chocolate, 18 g Pistachios, 9 g Pomelo, 150 g Salted chips, 15 g Salt, 2 g	Chicken eggs, 112 g Cocktail tomatoes, 80 g Graham roll, 65 g Arugula, 20 g Milk 1.5% fat, 10 g Plant-based butter, 5 g Pepper, 1 g Salt, 2 g
Dinner	Natural cottage cheese, 200 g Tomato, 120 g Chives, 5 g Black pepper, 1 g White salt, 1 g Water, 500 mL	Wholegrain tortilla, 61 g Cream cheese, 10 g, Delicatessen chicken ham, 15 g, Salad mix, 80 g, Mozzarella cheese, 7 g, Water, 300 ml	Ciabatta, 50 g Red pesto, 20 g Parma ham, 15 g Arugula, 10 g Sun-dried tomatoes, 14 g	Boiled potatoes, 150 g Fresh cod, 200 g Sauerkraut salad, 178 g Salt, 2 g Natural rice cakes, 20 g Hummus, 20 g Radish, 60 g	Spinach, 25 g Orange, 100 g Water, 125 mL Banana, 80 g	Red bell pepper, 80 g Cucumber, 120 g Radish sprouts, 20 g Feta cheese, 60 g Rapeseed oil, 5 g Apple cider vinegar, 5 g Pepper, 1 g Oregano, 1 g Basil, 1 g

In addition to the listed fluids, mineral water was consumed regularly throughout the entire day. The total average amount of water consumed in the first stage was 2349.3 ± 226.65 L and in the second stage it was 3923.26 ± 508.02 L.

**Table 3:** Systolic and diastolic blood pressure values, pulse, average number of steps, and hours of sleep in stage I and stage II of the study.

	Stage I	Stage II	Student’s *t*-Test	
	Average and Standard Deviation	Average and Standard Deviation	*t*	*p*
Systolic blood pressure, morning (mmHg)	94.57 ± 3.59	100.23 ± 5.42	−4.69	0.01
Systolic blood pressure, afternoon (mmHg)	94.57 ± 3.72	104.07 ± 4.19	−9.13	0.01
Systolic blood pressure, evening (mmHg)	92.13 ± 4.01	99.33 ± 4.89	−6.13	0.01
Systolic blood pressure, whole day (mmHg)	93.76 ± 3.86	101.21 ± 4.81	−11.41	0.01
Diastolic blood pressure, morning (mmHg) (g)	57.67 ± 2.08	59.27 ± 3.99	−1.91	0.01
Diastolic blood pressure, afternoon (mmHg)	58.13 ± 1.93	60.63 ± 3.14	−3.65	0.01
Diastolic blood pressure, evening (mmHg)	56.73 ± 6.81	58.4 ± 7.26	−0.90	0.01
Diastolic blood pressure, whole day (mmHg)	57.51 ± 2.99	59.43 ± 4.68	−3.26	0.01
Pulse, morning (bpm)	76.1 ± 5.37	76.87 ± 10.05	−0.36	0.81
Pulse, afternoon (bpm)	76.4 ± 7.65	75.7 ± 6.68	0.37	0.77
Pulse, evening (bpm)	73.57 ± 6.21	75.03 ± 7.11	−0.84	0.79
Pulse, whole day (bpm)	75.36 ± 6.6	75.87 ± 8.23	−0.46	0.86
Steps	4589.83 ± 852.38	8695.5 ± 1655.46	−11.87	0.01
Sleep (h)	5.83 ± 0.78	6.97 ± 0.95	−4.98	0.01

*t*: Student’s *t*-test value; *p*: significance level, significant at *p* ≤ 0.05; bpm: beats per minute (heart rate).

**Table 4:** Values of basic body composition parameters measured using the BIA method in the first and second stages of the study.

	Stage I	Stage II	Student’s *t*-Test	
	Average and Standard Deviation	Average and Standard Deviation	*t*	*p*
Body mass (kg)	48.15 ± 0.46	47.63 ± 0.58	1.56	0.15
Fat mass (kg)	8.95 ± 0.43	8.67 ± 0.85	0.66	0.52
Muscle mass (kg)	37.2 ± 0.32	36.97 ± 0.37	1.07	0.31
Lean body mass (kg)	39.2 ± 0.32	38.63 ± 0.69	1.68	0.12
Water content (kg)	28.37 ± 0.46	28.4 ± 0.59	−0.10	0.92
BMI (kg/m^2^)	18.12 ± 0.18	17.95 ± 0.24	1.26	0.24

*t*: Student’s *t*-test value; *p*: significance level, significant at *p* ≤ 0.05.

## Data Availability

The data used to support the findings of this study can be made available by the corresponding author upon request.
